# Restenosis after excimer laser coronary atherectomy and drug-coated balloon dilation in Takayasu’s arteritis: a case report and review of the literature

**DOI:** 10.1186/s12959-023-00529-9

**Published:** 2023-08-10

**Authors:** Shichu Liang, Jinming Yang, Min Ma, Minggang Zhou, Zhiyue Liu, He Huang, Yong He

**Affiliations:** 1 Department of Cardiology, West China Hospital, Sichuan University, No.37 Guoxue Alley, Chengdu, 610041 China; 2 Department of Rheumatology and Immunology, West China Hospital, Sichuan University, Chengdu, China

**Keywords:** Takayasu’s arteritis, Excimer laser coronary atherectomy, Drug-coated balloon, Anti-inflammation

## Abstract

Takayasu’s arteritis (TAK) is a rare chronic granulomatous arteritis that mainly affects the aorta and its major branches. Coronary artery (CA) involvement can be observed in 10–25% of TAK patients. We report a 21-year-old young female who was previously diagnosed with TAK and severe left main coronary artery (LMCA) stenosis and underwent numerous percutaneous coronary interventions (PCIs) in our hospital due to in-stent restenosis (ISR). This time, an excimer laser coronary atherectomy (ELCA) and drug-coated balloon (DCB) dilation was taken at the LMCA for the ISR. The blood flow was smooth after the operation, and she was symptom-free after discharge. Unfortunately, 5 months later, severe intimal hyperplasia was still seen in the stent of LMCA and left anterior descending (LAD) coronary artery. A coronary artery bypass graft surgery (CABG) was performed, and she has been symptom-free ever since. ELCA plus DCB is one of the novel ways we first reported. However, ensuring long-term inflammation control is equally important to restore blood flow. The combination of revascularization and anti-inflammation/immunosuppression is recommended to improve the outcomes of TAK patients with CA involvements.

**Supplementary Information:**

The online version contains supplementary material available at 10.1186/s12959-023-00529-9.

## Introduction

Takayasu’s arteritis (TAK), characterized by involvement of the aorta and its major branches, is a rare chronic granulomatous arteritis commonly found in children and young adults [1, 2]. The pathological presentation of TAK includes systemic chronic inflammatory processes in the blood vessels [2]. Vascular stenosis occurs in about 90% of TAK patients at the chronic stage of the disease, affecting the cerebral vasculature and the limbs primarily [3]. Coronary artery (CA) involvement can only be observed in 10–25% of TAKs [4]. Although stenotic CAs are rare, adverse events (including myocardial ischemia and myocardial infarction) often result in poor outcomes for patients with TAK. Therefore, it has become one of the essential focus on treating TAK with CA involvement.

Treatment strategies for TAK with CA involvement include revascularization, and anti-inflammatory and/or immunosuppressive therapies. Despite the absence of significant differences between medical therapy and revascularization in cardiac mortality [5], emergency revascularization therapy is urgently needed when TAK patients suffer from acute myocardial infarction [6], as drugs cannot restore coronary blood flow in the acute phase.

Revascularization approaches include percutaneous coronary interventions (PCI) and coronary artery bypass graft surgery (CABG), and the former is favorable by patients due to minimal invasion and comparable cardiac mortality [5]. PCIs include metallic stent implantation and balloon dilatation, but in-stent restenosis (ISR) frequently occurs in metallic stent implantation. For ISR management, drug-eluting stents (DES), drug-coated balloons (DCB), and excimer laser coronary atherectomy (ELCA) are currently used [7]. Only a few studies are currently evaluating DES/DCB for treating patients with TAK and CA involvements, and the effectiveness is unclear. Here, we reported the first case in which we combined ELCA and DCB dilation in a TAK female suffering from repeated ISRs, and performed a literature review of TAK cases with CA involvements undergoing PCI treatment.

## Case presentation

A 21-year-old TAK female with a history of repeated stent implantation presented to our hospital in August 2021, complaining of 5-day chest pain. This was her fifth hospitalization after the first in January 2018.

She was first admitted to our hospital in January 2018 for chest pain. The patient proclaimed no skin lesions, arthralgia, or recurrent oral ulcers. Furthermore, she had no family history of congenital heart disease. Physical examinations showed that asymmetric blood pressure in the upper limbs with differences was greater than 20 mmHg. No obvious murmurs were detected upon auscultation of the cardiac valve areas, bilateral carotid arteries, subclavian arteries, and renal arteries. Laboratory examination showed troponin T level increased to 28.6 ng/L (upper limit of normal [ULN]: 14 ng/L). The level of erythrocyte sedimentation rate (ESR) (10.0 mm/h, ULN: 26 mm/h) and C-reactive protein (CRP) (3.97 mg/L, ULN: 5 mg/L) were normal. Anti-nuclear antibodies, anti-neutrophil cytoplasmic antibodies, anti-cyclic citrullinated peptide antibodies, rheumatoid factor, and serologic tests for syphilis were all negative. The ultrasound of the cervical arteries showed uneven diameter and thickened wall of the right common carotid artery. The echocardiography indicated no abnormalities in the cardiac structures and wall motions. The enhanced-contrast computed tomography (CT) angiography indicated thickening of the vessel wall and mild narrowing in the proximal, middle, and distal segments of the right carotid artery (Supplementary Figure S1). No abnormalities were observed in other vessels.

She consented to the first coronary artery angiography (CAG), which revealed 90% stenosis of the left main coronary artery (LMCA) without stenosis of other CAs. A DES (3.5 × 12 mm, PROMUS Premier, Boston Scientific) was implanted in the LMCA. In the aftermath of the procedure, chest pain was relieved. She was diagnosed with TAK based on the 1990 American College of Rheumatology criteria [8]. Glucocorticoids, mycophenolate mofetil, aspirin, and clopidogrel were prescribed to the patient and maintained after discharge. Due to her financial constraints, biologics was declined to use. The medications were modified in the outpatient rheumatology department.

**Fig. 1:**
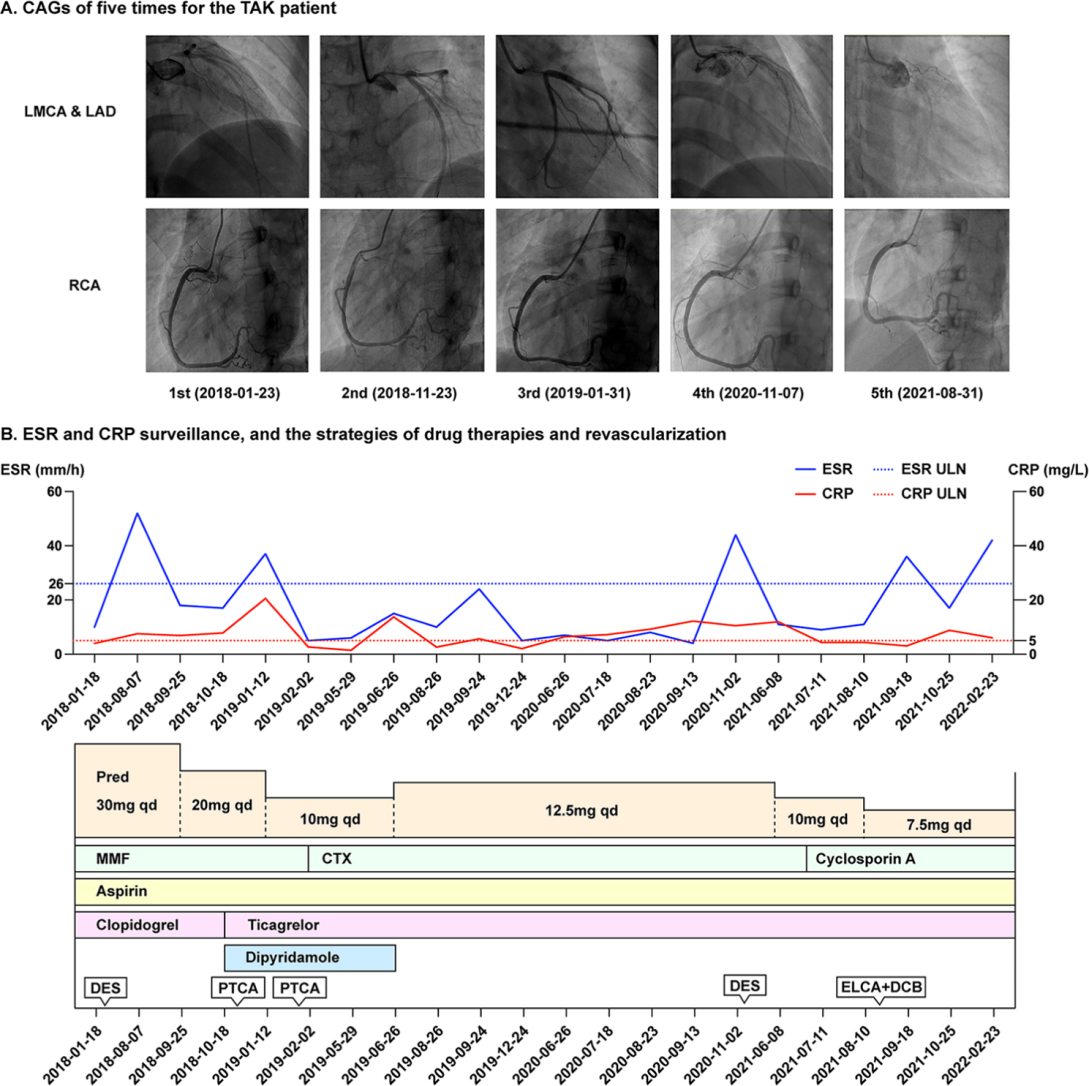
The clinical course of the disease. After the first hospitalization, the second to fourth coronary artery angiographies (CAGs) showed in-stent restenosis in the left main coronary artery (LMCA) and new stenosis of the left anterior descending (LAD) and left circumflex artery. Percutaneous transluminal coronary angioplasties were performed in the second to third hospitalization, and another drug-eluting stent (4.0 × 12 mm, PROMUS Element, Boston Scientific) was implanted in conjunction of LAD and LMCA in the fourth admission (November 2020). She consistently received anti-inflammation therapy and glucocorticoids tapered at 10 mg daily orally. Mycophenolate mofetil was changed to cyclophosphamide and successively cyclosporine A due to the twice bounce of erythrocyte sedimentation rate and C-reactive protein when in the outpatient of the rheumatology department. She took excimer laser coronary atherectomy and drug-coated balloon dilation in the fifth hospitalization. (**A.** CAGs of five times for the Takayasu’s arteritis patient; **B**. Erythrocyte sedimentation rate and C-reactive protein surveillance, and drug therapies)

**Fig. 2:**
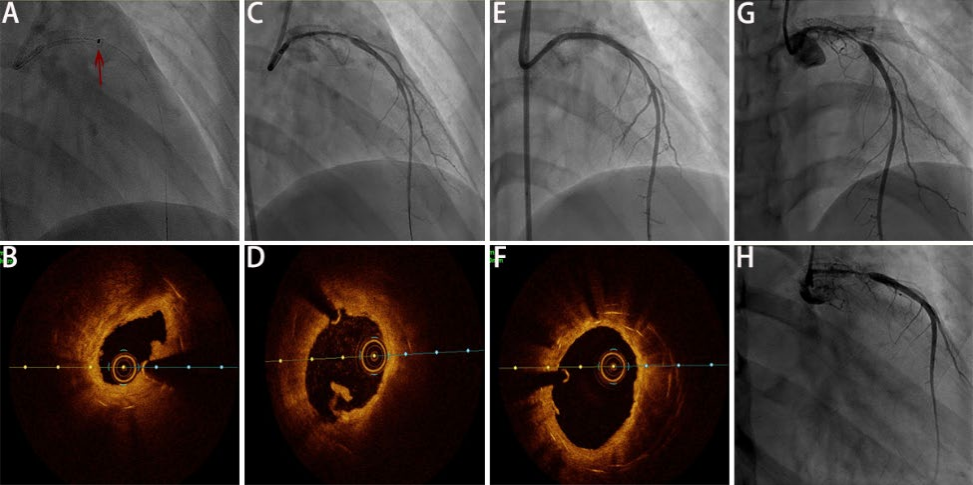
Coronary angiography and optical coherence tomography of coronary lesions during the fifth hospitalization (**A–B**: Before excimer laser coronary atherectomy, the minimal luminal area was 2.8 mm^2^; **C–D**: After excimer laser coronary atherectomy, the minimal luminal area was 5.1 mm^2^; E–F: After drug-coated balloon dilation, the minimal luminal area was 6.7 mm^2^; G–H: Five-month follow-up. Red arrow: laser catheter)

She took the medication irregularly regardless of the prescription, and the disease activity were not persistently controlled (based on Kerr’s criteria [9] and Indian Takayasu Clinical Activity Score [ITAS] [10], shown in the Supplementary Table S1). She suffered from recurrent chest pain during follow-up and was admitted three times after the first DES implantation (Fig. 1). In this admission in August 2021, glucocorticoids and cyclosporine A kept the levels of ESR and CRP relatively normal. Considering the history of multiple stent implantations and ISRs and her refusal of CABG, ELCA and DCB dilation was performed at the LMCA after obtaining the informed consent of the patient. ELCA utilized an incremental energy setting, starting with 40 mJ/40Hz and completing with 60 mJ/40Hz. After laser atheroablation, a 3.5 × 30 mm paclitaxel-coated balloon (Braun Melsungen, AG Vascular Systems, Berlin, Germany) was used. After the operation, the blood flow was smooth (Fig. 2. A–F). The results of CA involvements and medication prescriptions were shown in Supplementary Table S2.

Although she was symptom-free after discharge and was prescribed continually with glucocorticoids, cyclosporine A, aspirin, and ticagrelor, unfortunately, the follow-up CAG after five months showed severe intimal hyperplasia in the stents of LMCA-LAD conjunction, with maximum stenosis of approximately 90% (Fig. 2. G-H). A CABG was eventually performed, and she has been symptom-free ever since.

## Discussion

This is the first case of ELCA plus DCB dilation in a TAK female with CA involvement after repeated ISRs, with long-term anti-inflammation and immunosuppression therapies. Chest pain was controlled in the acute phase but she was treated with CABG due to ISRs after the ELCA plus DCB. This case suggested several important points on TAK with CA involvements. Firstly, the diagnosis of TAK should be established in the early stage because it determined whether anti-inflammation and immunosuppression is needed. Secondly, it is equally important for a TAK patient with CA involvements to restore blood flow of CA in the acute phase and to ensure inflammation control in the long-term follow-up.

When young patients suffer from acute chest pain without a family history or a personal history of cardiovascular disease, the evaluation of peripheral arteries, autoantibodies, and CAs is important in the diagnosis of TAK [11], because it determines that the patients with CA involvement need systematic anti-inflammatory/immunosuppressive therapies combined with local revascularization. The treatment of TAK patients usually differs from those only with coronary artery disease, as the latter often do not need anti-inflammation and immunosuppression therapy.

It is essential for patients with TAK who suffer from CA involvement to undergo revascularization, especially during the acute phase. However, the risk of developing ISR and undergoing re-revascularization is higher in PCI patients [5]. In our literature review (Table 1 and Supplementary Table S3), nearly 30% patients treated with bare metal stent (BMS) experienced revascularizations more than one times[12–15], whilst there were no patients treated with BMS among the groups with single revascularizations [16–24]. Currently, many cardiologists believe that TAK pathology is characterized by immune cell infiltration [24], and BMS is a permanent foreign material to the vessels, which may stimulate a rapid response to the non-self-substances and cause intimal hyperplasia and restenosis. Compared to BMS, DES has antiproliferative drugs coated on itself and can reduce inflammation-mediated intimal hyperplasia and treat local arterial inflammation. Previous studies have shown that DES has a lower ISR rate than BMS [25, 26]. However, despite the improvement in stent material and drug coating technologies, ISR incidences are still as high as 10%, whether with BMS or DES [27].

**Table 1:** Summary of reported cases of TAK with coronary involvement treated with drug-eluting stent and/or drug-coated balloon dilation

Literature	Age	Sex	Stenosis of coronary artery in CAG ^1^	Involved coronary artery and PCI or surgery ^1^	Pre-PCI medicine ^2^	Post-PCI medicine ^3^	PCI Follow-up duration ^4^	Symptoms after PCI	Follow-up CAG after PCI
**Furukawa Y, 2005** [12]	53	F	Stenosis of LMCA (90%) and distal bifurcation.	**The 1st PCI**: Ostial LMCA: bare metal stent; **The 2nd PCI**: LMCA-ISR: sirolimus-eluting stent.	Pred.	Not reported.	6 months.	Asymptomatic.	Minimal luminal narrowing in the LMCA and ostial LCX.
**Amir O, 2006** [16]	22	F	Stenosis of LMCA (60%).	Ostial LMCA: paclitaxel-eluting stent.	Anti-inflammatory therapy for 3 weeks (undetailed).	Steroid therapy.	3 months.	Asymptomatic.	An unimpeded stent without new lesions.
**Sakai H, 2006** [28]	37	F	Stenosis of LMCA.	**The 1st**: CABG; **Post-CABG PCI**: LMCA-LAD: zotarolimus-eluting stents; LMCA-LCX: zotarolimus-eluting stents.	Not reported.	Steroid therapy.	4 months.	Asymptomatic.	No new coronary lesions.
**Park JS, 2009** [17]	37	M	Stenosis of ostial LAD (95%) and LCX (95%) and RCA (99%).	Ostial LAD: paclitaxel-eluting stent; Ostial LCX: paclitaxel-eluting stent; Ostial RCA: paclitaxel-eluting stent.	Pred.	Pred.	6 months.	Asymptomatic.	A patent stent without new lesions.
**Lee K, 2010** [13]	35	F	**The 1st CAG**: Stenosis of LMCA and RCA; **The 2nd CAG**: ISR of ostial RCA.	**The 1st PCI**: Ostial LMCA: paclitaxel-eluting stent; RCA: bare metal stent; **The 2nd PCI**: ISR of RCA: sirolimus-eluting stent.	Not reported.	No use of immunosuppressants.	5 years.	Relapse of angina.	3rd CAG showing severe restenosis at LMCA and RCA. The patient took CABG.
**Terasawa A, 2010** [18]	66	F	Stenosis of LMCA at anastomosis to graft (history of Bentall operation).	Ostial LMCA: sirolimus-eluting stent.	No use of steroid therapy.	Steroid therapy.	12 months.	Asymptomatic.	No new coronary lesions.
**Lee HK, 2011** [14]	54	F	**The 1st CAG**:Stenosis of ostial LMCA (60%); **The 2nd CAG**: LMCA-ISR (75%); **The 3rd CAG**: Restenosis of the in-stent site (≥ 90%).	**The 1st PCI**: LMCA: a stent; **The 2nd PCI**: LMCA: paclitaxel-eluting stent; **The 3rd**: CABG.	No use of anti-inflammatory therapy.	No use of anti-inflammatory therapy.	3 months.	Asymptomatic after the 1st PCI. Chest pain and dyspnea after the 2nd PCI. Asymptomatic after CABG.	No new stenosis or graft stenosis 1 year after CABG.
**Cheng Z, 2011** [19]	27	F	Stenosis of LMCA (95%) and ostial RCA (90%).	Ostial LMCA: sirolimus-eluting stent.	Pred.	Not reported.	Not reported.	Asymptomatic.	Not reported.
**Yokota K, 2012** [29]	52	F	**The 1st CAG**: Stenosis of the proximal LAD; **The 2nd CAG**: ISR of LAD (75%); **The 3rd and 4th CAG**:Stenosis only in the stent site with no other plaque progression or new stenosis.	**The 1st PCI**: proximal LAD: sirolimus-eluting stent; **The 2nd PCI**: LAD-ISR: sirolimus-eluting stent; **The 3rd and 4th PCI**: no stent implantation.	Steroid therapy (The drug was withdrawn due to the inactivity of the arteritis).	Steroid therapy (starting after the 4th PCI).	About 2 years.	Repeated chest pain during 1st to 4th PCI.	Patent stent site.
**Isser HS, 2013** [20]	15	F	Stenosis of ostial LMCA (90%) and normal LAD, LCX and RCA.	Ostial LMCA: zotarolimus-eluting stent.	Not reported.	Steroid therapy.	1 year.	Asymptomatic.	A patent stent in LMCA.
**Soeiro Ade M, 2013** [15]	33	F	**The 1st and 2nd CAG**:Not reported; **The 3rd CAG**: LAD-ISR (75%).	**The 1st PCI**: LMCA: bare metal stent; **The 2nd PCI**: sirolimus-eluting stent; **The 3rd**: CABG.	MTX and Pred.	Pred, MMF and chloroquine after CABG.	18 months.	Chest pain after 2nd PCI. Asymptomatic after CABG.	No new stenosis or graft stenosis.
**Camuglia AC, 2015** [21]	21	F	Stenosis of LMCA with a normal RCA.	Ostial LMCA: bioresorbable vascular scaffold.	Not reported.	Pred and Aza.	8 months.	Asymptomatic.	No new coronary lesions.
**Rigatelli G, 2016** [22]	41	F	Stenosis of LMCA and RCA.	Ostial LMCA: paclitaxel-eluting stent; RCA: paclitaxel-eluting stent.	Not reported.	Steroid therapy.	Not reported.	Asymptomatic.	Not reported.
**Empen K, 2017** [30]	24	F	**The 1st CAG**: Stenosis of LMCA; **The 2nd CAG**: LMCA-ISR.	**The 1st PCI**: Ostial LMCA: everolimus-eluting stent; **The 2nd**: CABG.	Not reported.	Pred and CTX (after 1st PCI); Pred and tocilizumab (after 2nd CAG and before CABG).	Not reported.	Recurrent angina after 1st PCI. Asymptomatic after CABG.	Not reported.
**Macedo LM, 2019** [31]	23	F	**The 1st CAG**: Stenosis of LAD and LCX; **The 2nd CAG**: Stenosis of LMCA and venous graft (70%) with normal arterial graft.	**The 1st**: CABG; **Post-CABG PCI**: Venous graft-LAD: drug-eluting stent.	Not reported.	Pred and MTX after CABG; Not reported after PCI.	Not reported.	Chest pain after CABG. Asymptomatic after post-CABG PCI.	Not reported.
**Sammel AM, 2019** [32]	55	F	**The 1st CAG**: Stenosis of ostial LAD (99%) and RCA (100%); **The 2nd CAG**: ISR of ostial LAD (90%); **The 3rd CAG**: Stenosis of LAD and venous graft (95%).	**The 1st PCI**: LAD: zotarolimus-eluting stent; **The 2nd**: CABG.	Not reported.	DAPT after the 1st PCI; Immunosuppressive therapy with Pred and MTX 6 months after CABG.	36 months.	Chest pain after the first PCI and CABG. Asymptomatic after immunosuppressive Drugs.	Not reported.
**Shimizu T, 2020** [33]	55	F	**The 1st CAG**: Stenosis of ostial LAD (90%) and LCX (99%); **The 2nd CAG**: Restenosis of ostial LAD, ostial LCX and LMCA.	**The 1st PCI**: Ostial LAD: coronary atherectomy catheter and paclitaxel-coated balloon; **The 2nd**: CABG.	Not reported.	Pred after CABG.	12 months.	Asymptomatic after CABG and Pred prescription.	Not reported.
**Madhavan MV, 2020** [34]	17	F	**The 1st CAG**: Stenosis of LMCA, ostial RCA (60%), and right-to-left collaterals; **The 2nd CAG**: Stenosis of SVG-RCA, LIMA-obtuse marginal (70 and RIMA-LAD (70%).	**The 1st**: CABG; **Post-CABG PCI**: LMCA: zotarolimus-eluting stents.	Steroid therapy.	Pred, MTX and tocilizumab.	6 months.	Recurrent chest pain after CABG. Asymptomatic after post-CABG PCI.	No performance due to the patient’s refusal.
**Zhou S, 2021** [23]	22	F	Stenosis of ostial LMCA and RCA.	Staged PCI: RCA: sirolimus-eluting stent.	Not reported.	Pred and MTX.	15 months.	Asymptomatic.	Regression of ostial LMCA and a patent RCA stent
**Chiew KLX, 2021** [24]	47	F	Stenosis of LAD and LMCA.	Proximal LAD to LMCA: drug-coated balloon.	Aza.	Aza first. After the follow-up CAG, Pred, and tocilizumab were used, but lastly, only MTX.	4 months.	Asymptomatic.	No new stenosis.
**Chen Q, 2022** [35]	41	F	**The 1st CAG**: Stenosis of ostial LMCA (40%), LAD (75% in proximal segment and 90% in middle segment), LCX (80%) and ostial RCA (90%); **The 2nd CAG**: Stenosis of ostial LMCA (75%) with patent stent in LAD.	**The 1st PCI**: LAD: zotarolimus-eluting stent; RCA: zotarolimus-eluting stent.	Not reported.	Steroid therapy was stopped until there was no increase in CRP and ESR.	Not reported.	Chest discomfort after the 1st CAG, and she refused post-PCI CABG.	Not reported.

^1^ CAG and PCI represent the first coronary angiography and percutaneous coronary intervention after the first admission; ^2^ Pre-medicine refers to the use of anti-inflammatory/ immunosuppressive drugs before DES/DCB; ^3^ Post-medicine refers to the use of anti-inflammatory/ immunosuppressive drugs after DES/DCB; ^4^ The time refers to the interval between follow-up CAG after DES/DCB.

**Abbreviation**: F: female; M: male; Aza: azathioprine; BMS: bare-metal stent; CABG: coronary artery bypass graft surgery; CAG: coronary artery angiography; CRP: C-reactive protein; CTX: cyclophosphamide; DES: drug-eluting stent; ESR: erythrocyte sedimentation rate; LAD: left anterior descending; LMCA: left main coronary artery; LCX: left circumflex artery; LIMA: left internal mammary artery; MMF: mycophenolate mofetil; MTX: methotrexate; PCI: percutaneous coronary interventions; Pred: prednisolone; RCA: right coronary artery; SVG: saphenous vein graft

DCB is a novel technology for ISR treatment and it ensures coated antiproliferative drugs diffuse into culprit CAs, inhibiting neointimal hyperplasia. The DCB does not place an external object inside the vessels as the DES does, therefore it does not stimulate inflammatory responses by metal trabeculae and polymers in the endothelial and smooth muscles of the vessels [36]. However, a meta-analysis found that DCB has a higher rate of revascularization of the target vessel three years after PCI than DES, although the incidence rates were comparable [37]. The findings do not appear to apply to CA involvements related to TAK. Chiew et al. [24] reported a 47-year-old TAK woman with CA involvement treated with the DCB. Four months after DCB dilation, optical coherence tomography revealed complete endothelial healing, which implies luminal enlargement from positive remodeling [24]. However, the specific effectiveness of DCB on TAK-related CA involvement is limited by a small number of cases reports and the absence of large-scale studies and prospective trials.

ELCA, a crucial way of revascularization in our case, is feasible and has been applied in various complicated CA lesions, such as thrombus, repeated ISR, and under-expansion of the stent [38, 39]. Our case is the first application of ELCA before DCB dilation in patients with TAK with CA involvement. After ELCA and DCB dilation, no acute chest pain was observed for our patient during the next five months, which suggested that the combined revascularization strategies without implanting a permanent foreign body could be a viable option for these patients. However, a post-DCB CAG revealed another ISR despite being symptom-free. Nevertheless, it should be noted that the damage to the coronary intima associated with ELCA might contribute to stent thrombosis and restenosis [40]. Therefore, despite the potential feasibility of ELCA in TAK with CA involvements, the conservation of coronary intima during ELCA is critical to preventing ISR in the long-term follow-up. Our patient eventually underwent CABG and has been asymptomatic since then, indicating that CABG might be the best way to solve ISR in TAK with CA involvements so far [41].

In addition to local revascularization or non-implantation intervention, persistent disease activity is strongly related to ISR after PCIs in TAK patients, highlighting the importance of long-term anti-inflammation therapy [6]. Immunosuppressants include traditional drugs such as leflunomide, cyclophosphamide, and methotrexate [42, 43] and biologics such as Tocilizumab [44]. Steroids are the baseline medication of TAK. But almost 29–73% of TAK patients also required additional immunosuppressants to induce or maintain remission and reduce the side effect of glucocorticoids [45]. The long-term administration of glucocorticoids may lead to dyslipidemia [46], posing a potential risk of aggravating CA lesions. In our case, statin therapy was prescribed to the patients and the lipid levels have been fluctuating within the normal range (Supplementary Table S4). The progression of the CA lesions of our case is more likely associated with inadequate control of inflammation. Therefore, anti-inflammatory therapy is essential to reduce the risk of ISR [47]. Other two cases in the literature review also showed repeated ISR after stent implantation without anti-inflammatory or immunosuppressants [13, 14]. In a retrospective study with 48 TAK patients, those who continued maintenance therapy with low-dose steroids had a lower incidence of ISR [48], which suggested that maintenance of anti-inflammatory treatment is vital to decrease the risk of ISRs.

As for immunosuppressants, Sammel et al. [32] reported a 55-year-old woman who underwent treatment for critical coronary stenosis using BMS, DES, and eventually CABG, but suffered from restenosis after each procedure. After the patient was given immunosuppressants, the involved blood vessel became largely improved. Additionally, regression of the ostial lesions was observed in a TAK patient who received immunosuppressive treatment [49]. These cases highlighted the significance of immunosuppressive therapies in TAK patients with CA involvement, but there is no relevant research to determine whether TAK patients need additional oral or intravenous immunosuppressants to prevent ISR after DES/DCB, because several coated drugs on DES/DCB (including paclitaxel, zotarolimus, and sirolimus) have a local immunosuppressive effect. Interleukin-6 receptor blocker (such as Tocilizumab) and tumor necrosis factor inhibitors now are considered as a treatment option for TAK [24, 30, 34, 50]. TAK patients scheduled for CABG may also benefit from tocilizumab for the per-operative period [51]. However, our patient declined biologics due to financial constraints.

## Conclusion

In summary, the case report and literature review suggested that the diagnosis of TAK at the early stage is essential due to its determination on the use of anti-inflammatory and immunosuppressive therapies. The strategy without intravascular object implantation may be a feasible way for revascularization in the acute phase of TAK patients with CA involvements. ELCA plus DCB is one of the novel ways we first reported. However, ensuring long-term inflammation control is equally important to restore the blood flow. The combination of revascularization and anti-inflammation/immunosuppression is recommended to improve the outcomes of TAK patients with CA involvements. More research is warranted on the novel revascularization strategies and therapies for the TAK patients.

### Electronic supplementary material

Below is the link to the electronic supplementary material.

**Supplementary Material 1. Table S1**. The Kerrs Score, ITAS Score and ITAS. A Score during follow-up. **Table S2**. The results of the CAG of our patient. **Table S3**. The manufacturers of the stents and balloons. **Table S4**. Fluctuation of the platelet count and serum lipid profiles of the whole process. **Table S5**. Search strategies for searching case reports of Takayasu arteritis patients with coronary artery involvement. **Figure S1**. The cervical artery CTA images of the patient.

## Data Availability

The data presented in this study are available on reasonable request from the corresponding author.

## Abbreviations

BMS: bare metal stent
CA: coronary artery
CAG: coronary artery angiography
CABG: coronary artery bypass graft surgery
CRP: C-reactive protein
CT: computed tomography
CTX: cyclophosphamide
DCB: drug-coated balloons
DES: drug-eluting stents
ELCA: excimer laser coronary atherectomy
ESR: erythrocyte sedimentation rate
ISR: in-stent restenosis
LAD: left anterior descending artery
LCX: left circumflex artery
LMCA: left main coronary artery
MMF: mycophenolate mofetil
TAK: Takayasu’s arteritis
PCI: percutaneous coronary intervention
Pred: prednisolone
RCA: right coronary artery
