# Supplementary material for: Restenosis after excimer laser coronary atherectomy and drug-coated balloon dilation in Takayasu’s arteritis: a case report and review of the literature
Source: Thromb J. 2023 Aug 10;21:87. doi: 10.1186/s12959-023-00529-9 (PMC10413599; doi:10.1186/s12959-023-00529-9)
Supplement: Supplementary file 1 — Supplementary Material 1. Table S1. The Kerrs Score, ITAS Score and ITAS. A Score during follow-up. Table S2. The results of the CAG of our patient. Table S3. The manufacturers of the stents and balloons. Table S4. Fluctuation of the platelet count and serum lipid profiles of the whole process. Table S5. Search strategies for searching case reports of Takayasu arteritis patients with coronary artery involvement. Figure S1. The cervical artery CTA images of the patient. [file 12959_2023_529_MOESM1_ESM.docx]

**Supplementary Table S1. The Kerr’s Score, ITAS Score and ITAS.A Score during follow-up.**

|  | **Kerr Score** | **Active^*^** | **ITAS Score** | **Active^**^** | **ITAS.A Score** | **Active^***^** | **Glucocorticoid dose** |
| --- | --- | --- | --- | --- | --- | --- | --- |
| **20180118** | 2 | Yes | 3 | Yes | 3 | No | 30mg qd |
| **20180807** | 3 | Yes | 3 | Yes | 6 | Yes | 30mg qd |
| **20180925** | 0 | No | 1 | No | 2 | No | 20mg qd |
| **20181018** | 2 | Yes | 3 | Yes | 4 | No | 20mg qd |
| **20190112** | 3 | Yes | 3 | Yes | 7 | Yes | 20mg qd |
| **20190202** | 0 | No | 1 | No | 1 | No | 10mg qd |
| **20190529** | 0 | No | 1 | No | 1 | No | 10mg qd |
| **20190626** | 0 | No | 1 | No | 3 | No | 10mg qd |
| **20190826** | 0 | No | 1 | No | 1 | No | 12.5mg qd |
| **20190924** | 1 | No | 1 | No | 2 | No | 12.5mg qd |
| **20191224** | 0 | No | 1 | No | 1 | No | 12.5mg qd |
| **20200626** | 0 | No | 1 | No | 2 | No | 12.5mg qd |
| **20200718** | 0 | No | 1 | No | 2 | No | 12.5mg qd |
| **20200823** | 0 | No | 1 | No | 2 | No | 12.5mg qd |
| **20200913** | 0 | No | 1 | No | 3 | No | 12.5mg qd |
| **20201102** | 3 | Yes | 3 | Yes | 7 | Yes | 12.5mg qd |
| **20210608** | 0 | No | 1 | No | 3 | No | 12.5mg qd |
| **20210711** | 0 | No | 1 | No | 1 | No | 10mg qd |
| **20210810** | 2 | Yes | 3 | Yes | 3 | No | 10mg qd |
| **20210918** | 2 | Yes | 1 | No | 2 | No | 7.5mg qd |
| **20211025** | 0 | No | 1 | No | 2 | No | 7.5mg qd |
| **20220223** | 2 | Yes | 1 | No | 4 | No | 7.5mg qd |

**^*^Kerr’s Score≥2 was considered as active;**

**^**^ITAS Score≥2 was considered as active;**

**^***^ITAS.A Score≥5 was considered as active.**

**Supplementary Table S2. The results of the CAG of our patient.**

| **Time** | | **Degree of stenosis in CAG** | | | | **PCI strategies** | **Post-PCI therapy** | |
| --- | --- | --- | --- | --- | --- | --- | --- | --- |
|  |  | **LMCA** | **LAD** | **LCX** | **RCA** |  | **Anti-platelet therapy** | **Anti-inflammatory/**  **immunosuppressive therapy** |
| **1^st^** | **2018.01.23** | 90% | 0% | 0% | 0% | LMCA: 3.5 mm×12 mm×1 stent (PROMUS Premier, Boston Scientific). | Aspirin 100 mg qd; Clopidogrel 75 mg qd | Prednisone 30 mg qd; Mhenolate mofetil 750 mg bid |
| **2^nd^** | **2018.11.23** | 99% | 60% | 0% | 0% | LMCA-LAD conjunction: PTCA | Aspirin 100 mg qd; Ticagrelor 90 mg bid | Prednisone 20 mg qd; Mhenolate mofetil 750 mg bid |
| **3^rd^** | **2019.01.31** | 99% | 60% | 0% | 0% | LMCA-LAD conjunction: PTCA | Aspirin 100 mg qd; Ticagrelor 90 mg bid; Dipyridamole 50 mg qd | Prednisone 10 mg qd; Cyclophosphamide 50 mg qd |
| **4^th^** | **2020.11.07** | 90-95% | 90-95% | 95% | 0% | LMCA-LAD conjunction: 4.0 mm×12 mm×1 stent (PROMUS Element, Boston Scientific) | Aspirin 100 mg qd; Ticagrelor 90 mg bid; | Prednisone 10 mg qd; Cyclophosphamide 50 mg qd |
| **5^th^** | **2021.08.31** | 99% | 99% | 90% | 50% | LMCA-LAD conjunction: laser catheter (Spectranetics, Philips Medical Systems, Inc.) + 3.5 mm×30 mm×1 paclitaxel-coated balloon (Braun Melsungen, AG Vascular Systems, Berlin, Germany). | Aspirin 100 mg qd; Ticagrelor 90 mg bid; | Prednisone 10 mg qd; Cyclosporin A 75 mg qd |
| **6^th^** | **2022.03.20** | 99% | 99% | 90% | 60% | Refuse PCI and CABG were taken. | Aspirin 100 mg qd; Ticagrelor 90 mg bid; | Prednisone 10 mg qd; Cyclosporin A 75 mg qd |

**Abbreviation:** LAD: left anterior descending; LCMA: left main coronary artery; LCX: left circumflex artery; PCI: percutaneous coronary interventions; RCA: right coronary artery.

**Supplementary Table S3. The manufacturers of the stents and balloons.**

| **Literature** | **Stents** |
| --- | --- |
| **Furukawa Y, 2005 [1]** | The 1st PCI: Ostial LMCA: bare metal stent (3.0×15 mm ACS RX Multi-Link™, Guidant);  The 2nd PCI: Ostial LMCA-ISR: sirolimus-eluting stent (3.5×23 mm Cypher, Johnson & Johnson). |
| **Amir O, 2006 [2]** | Ostial LMCA: paclitaxel-eluting stent (3.5×8 mm, TAXUS, Boston Scientific, Natick, Mass). |
| **Sakai H, 2006 [3]** | LMCA-LAD: Sirolimus-Eluting Stent (3.5×18 mm Cypher, Johnson & Johnson);  LMCA-LCX: Sirolimus-Eluting Stent (3.5×23 mm Cypher, Johnson & Johnson). |
| **Park JS, 2009 [4]** | Ostial LAD: paclitaxel-eluting stent (5×18 mm, TAXUS, Boston Scientific, USA);  LCX: paclitaxel-eluting stent (5×24 mm, TAXUS, Boston Scientific, USA);  Ostial RCA: paclitaxel-eluting stent (5×12 mm, TAXUS, Boston Scientific, USA). |
| **Lee K, 2010 [5]** | The 1st PCI: Ostial LMCA: paclitaxel-eluting stent (3.5×12mm, TAXUS, Boston Scientific, MA, USA); RCA: bare-metal stent (BMS, Express 4.5 × 16 mm, Boston Scientific, MA, USA);  The 2nd PCI: ISR RCA: sirolimus-eluting stent (3.5×23 mm, Cypher, Cordis, Miami Lakes, FL, USA). |
| **Terasawa A, 2010 [6]** | Ostial LMCA: sirolimus-eluting stent (3.5×18 mm Cypher, Cordis Corporation, Miami, FL, USA). |
| **Lee HK, 2011 [7]** | The 1st PCI: LMCA: a stent (PICO™ 4.0×10 mm stent, AMG);  The 2nd PCI: LMCA: paclitaxel-eluting stent (3.5×12 mm, TAXUS, Boston Scientific, MA, USA);  The 3rd: CABG without PCI. |
| **Cheng Z, 2011 [8]** | Ostial LMCA: sirolimus-eluting stent (3.5×13 mm, Cypher, Cordis Europa N.V. Roden, Netherlands)). |
| **Yokota K, 2012 [9]** | The 1st PCI: proximal LAD: sirolimus-eluting stent (3.5×18 mm, Cypher, Cordis Corporation, Bridgewater, NJ, USA);  The 2nd PCI: In-stent LAD: another drug-eluting stent (3.5×23 mm, Cypher, Cordis Corporation, Bridgewater, NJ, USA).  The 3rd and 4th PCI: no stent implantation. |
| **Isser HS，2013 [10]** | Ostial LMCA: zotarolimus eluting stent (3.5×12mm, Endeavor Drug-Eluting Coronary Stent, Medtronic). |
| **Soeiro Ade M, 2013 [11]** | The 1st PCI: LMCA: bare-metal stent (5×20 mm, undetailed).  The 2nd PCI: sirolimus-eluting stent (3.5×18 mm Cypher).  The 3rd: CABG. |
| **Camuglia AC, 2015 [12]** | Ostial LMCA: bioresorbable vascular scaffold: Absorb 3.5×12 mm (Abbott Vascular, Santa Clara, CA) bioresorbable vascular scaffold. |
| **Rigatelli G, 2016 [13]** | Ostial LMCA: paclitaxel-eluting stent (3×12mm, Promus Premier stent, Boston Scientific USA);  RCA: paclitaxel-eluting stent (2.75×15mm, Promus Premier stent, Boston Scientific USA). |
| **Empen K, 2017 [14]** | The 1st PCI: Ostial LMCA: everolimus-eluting stent (undetailed);  The 2nd: CABG. |
| **Macedo LM, 2019 [15]** | The 1st: CABG.  Post-CABG PCI: venous graft-LAD: drug-eluting stent (undetailed); |
| **Sammel AM, 2019 [16]** | Ostial LAD: zotarolimus-eluting stent (Endeavour 3 ×15 mm; Medtronic Vascular, Santa Rosa, CA, USA). |
| **Shimizu T, 2020 [17]** | Ostial LAD: coronary atherectomy catheter (ATHEROCUT™, Nipro Corporation, Japan) followed by paclitaxel-coated balloon (SeQuent® Please, B. Braun, Melsungen, Germany). |
| **Madhavan MV， 2020 [18]** | The 1st: CABG.  Post-CABG PCI: LMCA: zotarolimus-eluting stents (undetailed); |
| **Zhou S, 2021 [19]** | Staged PCI: RCA: sirolimus-eluting stent (Firehawk 3.0×13 mm, Shanghai MicroPort Medical Corporation, China). |
| **Chiew KLX， 2021 [20]** | Proximal LAD to LMCA: drug-coated balloon (3.0×20 mm SeQuent Neo, B Braun, Hesse, Germany). |
| **Chen Q, 2022 [21]** | The 1st PCI: LAD: zotarolimus-eluting stent (Resolute, 2.25×18 mm, Medtronic); RCA: zotarolimus-eluting stent (Resolute, 3.5×18 mm, Medtronic). |

**Abbreviation:** CABG: Coronary artery bypass graft surgery; LAD: left anterior descending; LCMA: left main coronary artery; LCX: left circumflex artery; RCA: right coronary artery; PCI: percutaneous coronary interventions; PTCA: percutaneous transluminal coronary angioplasty.

**Supplementary Table S4.** **Fluctuation of the platelet count and serum lipid profiles of the whole process.**

|  | **TG (nmol/L,**  **reference range 0.29-1.83 nmol/L)** | **TC (nmol/L,**  **reference range 2.8-5.7 nmol/L)** | **HDL (nmol/L,**  **reference range > 0.9 nmol/L)** | **LDL (nmol/L,**  **reference range <4 nmol/L)** |
| --- | --- | --- | --- | --- |
| **20180118** | 0.55 | 4.86 | 1.79 | 2.46 |
| **20180807** | 0.67 | 3.8 | 1.32 | 2.4 |
| **20180925** | 1.21 | 3.39 | 1.2 | 1.82 |
| **20181018** | 0.87 | 3.08 | 1.06 | 1.68 |
| **20190112** | 0.51 | 2.45 | 1.24 | 1.16 |
| **20190202** | 0.52 | 4.3 | 2.28 | 2 |
| **20190529** | 0.83 | 2.95 | 1.39 | 1.39 |
| **20190626** | 1.05 | 2.76 | 1.23 | 1.23 |
| **20190826** | 1.11 | 2.98 | 1.23 | 1.3 |
| **20200626** | 1.63 | 3.12 | 1.26 | 1.54 |
| **20200718** | 1.61 | 3.48 | 1.26 | 1.67 |
| **20200913** | 1.7 | 3.22 | 1.03 | 1.67 |
| **20201102** | 1.4 | 2.78 | 1.15 | 1.4 |
| **20210608** | 1.43 | 2.98 | 1.09 | 1.43 |
| **20210711** | 1.14 | 3.04 | 1.21 | 1.54 |
| **20210810** | 1.26 | 3.04 | 1.1 | 1.47 |
| **20210918** | 1.21 | 2.75 | 1 | 1.3 |
| **20210921** | 1.08 | 2.77 | 1.14 | 1.45 |
| **20211025** | 1.73 | 3.01 | 1.06 | 1.45 |
| **20220223** | 1.39 | 3.22 | 1.02 | 1.72 |

**
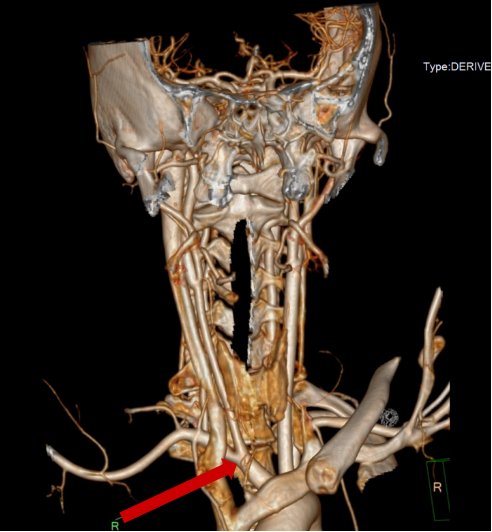
**

**Supplementary Figure S1. The cervical artery CTA images of the patient.** The right carotid artery in the proximal, middle, and distal segments reveal wall thickening and mild narrowing of the vessel lumen (red arrows). No abnormalities are observed in the remaining scanned area, including the neck and major intracranial arteries.

**Literature Research**

**Supplementary method**

The authors (S. Liang and J. Yang) independently searched the PubMed, EMBASE, and Web of science databases for articles published from inception until June 1, 2023, using the following heading terms: “Takayasu arteritis”, “coronary artery” and “case” (Supplementary Table S5). The search was carried out by combining subject words and free words. Only studies written in English were included. Relevant literature references were also searched to identify more eligible studies. The literature titles and abstracts were screened for primary screening, and then full-text acquisition and reading of the literature for rescreening were conducted. Cases without follow-up or details of the PCI were excluded.

Two independent readers (S. Liang and J. Yang) extracted the following information: first author, published year, age, sex, stenosis of coronary artery in coronary artery angiography (CAG), involved coronary artery (CA), Percutaneous coronary interventions (PCI) or surgery for the involved CA, pre-PCI medicine, post-PCI medicine, PCI Follow-up duration, symptoms and follow-up CAG after PCI. They were not blinded to the authors and institutions of included studies. Disagreements were resolved by a third reader (M. Ma), while Y. He and H. Huang supervised the entire process.

We analyzed the baseline characteristics of TAK patients who underwent PCIs and had involvement of the CAs. Due to the limited number of cases, we were unable to utilize the Cox proportional-hazards model in our analysis. Instead, we roughly calculated the rates of in-stent restenosis (ISR) and post-PCI coronary artery bypass grafting (CABG) in TAK patients with CA involvement.

**Supplementary Results**

Following the flow chart of the PRISMA for systematic literature search and study selection process (Supplementary Figure S1), we searched the databases including PubMed, EMBASE, and Web of Science, and initially yielded 259 literature citations, which were reduced to 70 after removing duplicates and irrelevant studies. A review of titles and keywords resulted in the exclusion of 74 studies, leaving 75 abstracts for evaluation by two authors (S. Liang and J. Yang). After full-text evaluation, 21 cases were selected. Clinical and angiographic features and outcomes of 22 patients (including our cases) are summarized in Table 1.

Cumulatively, there were 21 females (95.5%) among all the 22 cases, of whom the youngest patient was 15 [10] and the oldest patient was 66 [6] at the diagnosis. More than half of the patients (n = 13) was younger than 40 years old [2-5,8,10-12,14,15,18,19]. Ostial left main coronary artery (LCMA) and ostial left anterior descending (LAD) involvement prevailed among all the patients at the first CAG, and 11 (50.0%) patients suffered more than one CAs [4,5,8,12,13,15-19,21] at the first CAG. Nine patients (40.9%) underwent only one revascularization [2,4,6,8,10,12,13,19,20], among which there are 7 individuals treated with DES [2,4,6,8,10,13,19], 1 with bioresorbable vascular scaffold [12], and 1 with DCB [20]. Thirteen patients (59.1%) were treated with second revascularization after the first ones (both PCI or surgery) [1,3,5,7,9,11,14,15,16,17,18,21]. Among those who experienced revascularizations more than one times, 4 patients (4/13=30.7%) were implanted with BMS at the first revascularization [1,5,7,11], and 3 patients (3/13=23.1%) were treated with CABG first [3,15,18]. Six of the thirteen patients (46.2%) were eventuall treated with CABG owing to repeated restenosis [7,11,14,16,17].

As for anti-inflammatory drug therapies, the nine patients undergoing only one revascularization were all prescribed with pre-PCI and/or post-PCI steroid therapy and presented asymptomatic in the long-duration follow-up [2,4,6,8,10,12,13,19,20]. There were 2 other patients free of post-PCI anti-inflammatory medicines suffering stent implantation more than one times [5,7], but one still showed chest pain after the final revascularization [5]. Almost all patients with the combination of revascularization (one or more than one times) and anti-inflammatory/immunosuppressive therapies were asymptomatic [2-4,6,9-21]. No cardiovascular death was reported.

**Supplementary Table S5** Search strategies for searching case reports of Takayasu arteritis patients with coronary artery involvement.

| Databases | Number of articles retrieved | Search strategies |
| --- | --- | --- |
| Pubmed | 86 | #1 "Takayasu arteritis"[MeSH Terms] OR "Takayasu arteritis"[Title/Abstract])  #2 "coronary"[Title/Abstract] OR "coronary artery"[Title/Abstract]  #3 "case"[Title]  #4 #1 AND #2 AND #3 |
| Web of science | 102 | #1 TI=(Takayasu arteritis) OR AB=(Takayasu arteritis)  #2 TI=(coronary) OR AB=(coronary) OR TI=(coronary artery) OR AB=(coronary artery)  #3 TI=(case)  #4 #1 AND #2 AND #3 |
| EMBASE | 71 | #1 'Takayasu arteritis':ab,ti  #2 'coronary':ab,ti OR 'coronary artery':ab,ti  #3 'case':ti  #4 #1 AND #2 AND #3 |

**Reference**

1. Furukawa Y, Tamura T, Toma M, et al. Sirolimus-eluting stent for in-stent restenosis of left main coronary artery in takayasu arteritis. Circ J. 2005;69(6):752-755.
2. Amir O, Kar B, Civitello AB, Palanichamy N, Shakir A, Delgado RM 3rd. Unprotected left main stent placement in a patient with Takayasu's arteritis: an unusual solution for an unusual disease. Tex Heart Inst J. 2006;33(2):253-255.
3. Sakai H, Oyama N, Kishimoto N, et al. Revascularization of malignant coronary instent restenosis resulting from Takayasu's arteritis using sirolimus-eluting stents. Int Heart J. 2006;47(5):795-801. doi:10.1536/ihj.47.795
4. Park JS, Lee HC, Lee SK, et al. Takayasu's Arteritis Involving the Ostia of Three Large Coronary Arteries. Korean Circ J. 2009;39(12):551-555.
5. Lee K, Kang WC, Ahn T, et al. Long-term outcome of drug-eluting stent for coronary artery stenosis in Takayasu's arteritis. Int J Cardiol. 2010;145(3):532-535.
6. Terasawa A, Kondo K, Ishikawa S, Morimoto R, Tajika T, Hayashi Y. Sirolimus-eluting stent implantation for ostial stenosis of left main coronary artery after Bentall operation in aortitis syndrome. J Cardiol. 2010;55(1):147-150.
7. Lee HK, Namgung J, Choi WH, et al. Stenting of the Left Main Coronary Artery in a Patient With Takayasu's Arteritis. Korean Circ J. 2011;41(1):34-37.
8. Cheng Z, Yue C, Shen Z, Fang Q. Percutaneous coronary intervention in Takayasu's arteritis. Int J Cardiol. 2011;151(2):231-232.
9. Yokota K, Shimpo M, Iwata T, et al. A case of Takayasu arteritis with repeated coronary artery restenosis after drug-eluting stent implantation successfully treated with a combination of steroids. Intern Med. 2012;51(7):739-743.
10. Isser HS, Chakraborty P, Bansal S. Coronary angioplasty of left main coronary artery in patient with Takayasu's arteritis. Indian Heart J. 2013;65(5):650-652.
11. Soeiro Ade M, Pinto AL, Henares BB, Ribeiro HB, Lima FG, Serrano CV Jr. Takayasu arteritis: stenosis after bare-metal and drug-eluting stent implantation. Arq Bras Cardiol. 2013;100(1):e8-e11.
12. Camuglia AC, Randhawa VK, Lavi S. Takayasu arteritis involving the left main coronary artery treated with a bioresorbable vascular scaffold. Int J Cardiol. 2015;190:1-3.
13. Rigatelli G, Zuin M, Picariello C, Cardaioli P, Roncon L. Aortitis-related isolated bilateral coronary artery ostial stenosis in a young woman with acute coronary syndrome. Int J Cardiol. 2016;223:111-112.
14. Empen K, Hummel A, Beug D, Felix SB, Busch MC, Kaczmarek PM. Takayasu's arteritis: a case with relapse after urgent coronary revascularization. BMC Res Notes. 2017;10(1):311.
15. Macedo LM, Lima NA, de Castro Junior RL, Bannon SF. Takayasu arteritis with multiple coronary involvement and early graft relapse. BMJ Case Rep. 2019;12(4):e229383.
16. Sammel AM, Wolfenden HD, Joshua F, Jepson N. Reduced efficacy of transcatheter and surgical revascularization in Takayasu arteritis. Int J Rheum Dis. 2019;22(1):152-157.
17. Shimizu T, Sato A, Sakamoto K, et al. Intravascular ultrasound imaging of isolated and non aorto-ostial coronary Takayasu arteritis: a case report. BMC Cardiovasc Disord. 2020;20(1):260.
18. Madhavan MV, Coromilas EJ, Poterucha TJ, et al. Repeat Revascularization for Severe Recurrent Coronary Artery Disease in a Young Woman With Takayasu Arteritis. JACC Case Rep. 2020;2(1):77-81.
19. Zhou S, Gao C, Li F. Acute myocardial infarction with left main coronary artery ostial negative remodelling as the first manifestation of Takayasu arteritis: a case report. BMC Cardiovasc Disord. 2021;21(1):560.
20. Chiew KLX, Lim PO. Three-year outcome with drug-coated balloon percutaneous coronary intervention in coronary Takayasu arteritis: A case review. Catheter Cardiovasc Interv. 2021;97(5):841-846.
21. Chen Q, Zhao QM. Five-year outcome with revascularisation therapy in Takayasu arteritis with multiple coronary involvement: a case review. Cardiol Young. 2022;32(11):1857-1859.
